# Clinical Characteristics and Outcomes of Cardiogenic Shock in Acute Myocarditis Versus Takotsubo Cardiomyopathy: A Propensity Matched Nationwide Analysis

**DOI:** 10.1016/j.jscai.2026.105460

**Published:** 2026-06-18

**Authors:** Hossam Ibrahim, Oluchi Ndulue, Fatmaelzahraa Badr, Valentine C. Nriagu, Hasan Alarouri, Armando Seitllari, Binit Aryal, Supraja Achuthanandan, Steven Sorci, Bilal Malik, Gerald Hollander, Robert Frankel, Jacob Shani, Vijay Shetty

**Affiliations:** a Department of Internal Medicine, Maimonides Medical Center, Brooklyn, New York; b Department of Cardiology, Maimonides Medical Center, Brooklyn, New York

**Keywords:** cardiogenic shock, myocarditis, takotsubo cardiomyopathy

## Abstract

**Background:**

Cardiogenic shock (CS) is a life-threatening complication of acute myocarditis (MC) and takotsubo cardiomyopathy (TC). Comparative data on mechanical circulatory support (MCS) utilization and outcomes between these cohorts remain undefined.

**Methods:**

Using the National Inpatient Sample 2020-2022 database, adult hospitalizations with concomitant diagnoses of CS and MC (MC-CS) or TC (TC-CS) were identified. Patients with cardiac arrest, acute coronary syndrome, coronary revascularization, or incomplete records were excluded. A 1:1 propensity score match was performed to balance demographics, baseline comorbidities, and hospital characteristics. The primary outcome was in-hospital mortality. Secondary outcomes included MCS and resource utilization.

**Results:**

Among 7080 hospitalizations (MC-CS: n = 2825; TC-CS: n = 4255), patients with MC-CS were younger (46.5 vs 62.9 years) and predominantly male (58.0% vs 27.5%). Propensity score matching yielded 2435 balanced pairs. In the matched cohort, hospital mortality (26.5% vs 26.7%; *P* = .97) and length of stay (16.2 vs 16.0 days; *P* = .96) were comparable between groups. MC-CS was associated with higher use of temporary MCS (30.0% vs 10.5%; *P* < .001), including extracorporeal membrane oxygenation (14.2% vs 2.3%, *P* = .003), Impella (14.6% vs 6.8%, *P* = .04), and intra-aortic balloon pump (13.6% vs 3.1%, *P* = .003). Total hospital charges were higher for MC-CS ($435,121 vs $288,522; *P* = .008).

**Conclusions:**

In patients with CS, hospital mortality and length of stay are comparable between MC-CS and TC-CS when matched for baseline clinical risk. However, MC-CS involves nearly 3-fold higher utilization of MCS alongside higher hospital charges.

## Introduction

Cardiogenic shock (CS) is associated with a high in-hospital mortality rate of approximately 50%. Although acute myocardial infarction remains the leading cause of CS, advancements in the management of acute myocardial ischemia have led to the increasing recognition of nonischemic etiologies.^1^^,^^2^ Acute myocarditis (MC) and takotsubo cardiomyopathy (TC) are 2 common causes of de novo nonischemic CS that present as sudden-onset pump failure accompanied by troponin elevation and electrocardiographic changes that clinically mimic acute myocardial infarction. However, their underlying pathophysiology, clinical course, and outcomes are fundamentally distinct. MC is primarily driven by immune-mediated inflammatory myocardial injury, with presentations ranging from subacute heart failure to fulminant shock.^3^, ^4^, ^5^ Conversely, TC is a transient cardiomyopathy characterized by acute, reversible, stress-induced ventricular systolic dysfunction and apical ballooning, mediated by sympathetic catecholamine surges rather than obstructive coronary disease.^6^^,^^7^

Although they feature divergent pathophysiologies, both conditions can precipitate severe end-organ hypoperfusion and cellular hypoxia. CS complicates approximately 10% of both TC^8^, ^9^, ^10^, ^11^ and MC^12^^,^^13^ presentations, serving as a primary predictor of acute-phase mortality for both etiologies. In both clinical scenarios, temporary mechanical circulatory support (MCS) is increasingly deployed as a bridge to recovery, reducing myocardial workload and maintaining systemic perfusion.^2^

Despite their growing clinical relevance, comparative data evaluating the clinical trajectory and MCS utilization between these 2 distinct nonischemic cohorts are lacking. To our knowledge, no prior studies have directly compared CS secondary with MC vs TC. Therefore, the aim of this study is to evaluate the in-hospital course, MCS utilization, and clinical outcomes of patients presenting with MC-CS compared with TC-CS.

## Methods

### Data source

This retrospective cohort study used data from the National Inpatient Sample (NIS) for the years 2020 to 2022. The NIS, developed by the Healthcare Cost and Utilization Project and sponsored by the Agency for Healthcare Research and Quality, is the largest publicly available all-payer inpatient healthcare database in the United States. It contains data from approximately 7 million unweighted hospital stays annually, which, when weighted, estimate over 35 million hospitalizations nationally. Because the database contains fully deidentified patient information, this study was exempt from institutional review board approval.

### Study population and variable definitions

Using International Classification of Diseases, Tenth Revision, Clinical Modification (ICD-10-CM) codes (Supplemental Table S1), we identified all adult patients (aged ≥18 years) with a diagnosis of CS or use of MCS concomitant with either acute myocarditis (MC-CS) or takotsubo cardiomyopathy (TC-CS) at any diagnostic position. The cohort was stratified into 2 mutually exclusive groups based on etiology; patients with overlapping diagnoses were excluded to ensure distinct phenotypes. To strictly isolate nonischemic etiologies, we excluded patients with primary cardiac arrest, acute coronary syndrome (defined as acute myocardial infarction, angina, or acute ischemia at any diagnostic position), or those undergoing coronary revascularization via percutaneous coronary intervention or coronary artery bypass grafting. We further excluded interhospital transfers, elective admissions, and records with missing core demographic data. Baseline comorbidities were defined using the Charlson Comorbidity Index and Elixhauser comorbidity software, supplemented with specific diagnosis codes (Supplemental Table S1). In-hospital interventions were identified using ICD-10-Procedure Coding System codes, including invasive mechanical ventilation, hemodynamic monitoring via right heart catheterization (RHC), and MCS devices including micro-axial flow pumps, intra-aortic balloon pumps (IABP), veno-arterial extracorporeal membrane oxygenation (VA-ECMO), durable left ventricular assist devices, and orthotopic heart transplantation.

### Outcomes

The primary endpoint was in-hospital mortality. Secondary outcomes included MCS utilization, length of stay, total hospitalization charges, and discharge disposition. We also evaluated a composite of in-hospital complications, specifically acute liver injury, acute kidney injury, cerebrovascular accidents, intracranial and procedure-related bleeding, vascular complications, pericardial complications, and the need for new implantable cardioverter-defibrillator (ICD) implantation.

### Statistical analysis

Statistical analyses were conducted using Stata version 19.0 (StataCorp LLC). All statistical analyses were conducted using survey-specific commands to account for hospital-level clustering and stratification. Baseline characteristics were compared using a chi-square test for categorical variables, reported as weighted percentages, and survey-weighted linear regression for continuous variables, reported as mean ± standard error. To account for confounding, 1:1 propensity score matching was performed between MC-CS and TC-CS using a greedy nearest-neighbor algorithm without replacement. A caliper width of 0.1 pooled standard deviations of the propensity score was applied. The propensity score was estimated via logistic regression incorporating demographics (age, sex, race, median household income quartile, and primary expected payer), hospital characteristics (region, teaching status, and bed size), the Charlson Comorbidity Index, and specific comorbidities: coronary artery disease, prior myocardial infarction, prior percutaneous coronary intervention, prior coronary artery bypass grafting, chronic heart failure, peripheral vascular disease, chronic kidney disease, end-stage renal disease, obesity, diabetes mellitus, history of stroke, prior implantable cardioverter defibrillator, atrial fibrillation or flutter, dementia, and cancer. Covariate balance was assessed using standardized mean differences (SMDs), with an SMD ≤ 0.10 indicating adequate balance. Outcomes were compared using survey-weighted logistic and linear regression. A 2-sided *P* < .05 was considered statistically significant.

## Results

### Study population and incidence of cardiogenic shock

Using the 2020-2022 NIS, we identified 720,230 adult hospitalizations with a cardiogenic shock diagnosis (R57.0) or requiring MCS. To isolate a nonischemic CS cohort, we excluded cases involving acute ischemic events, coronary revascularization, and primary cardiac arrest. This resulted in a refined cohort of 285,925 nonischemic CS hospitalizations (Central illustration).

**Central Illustration:**
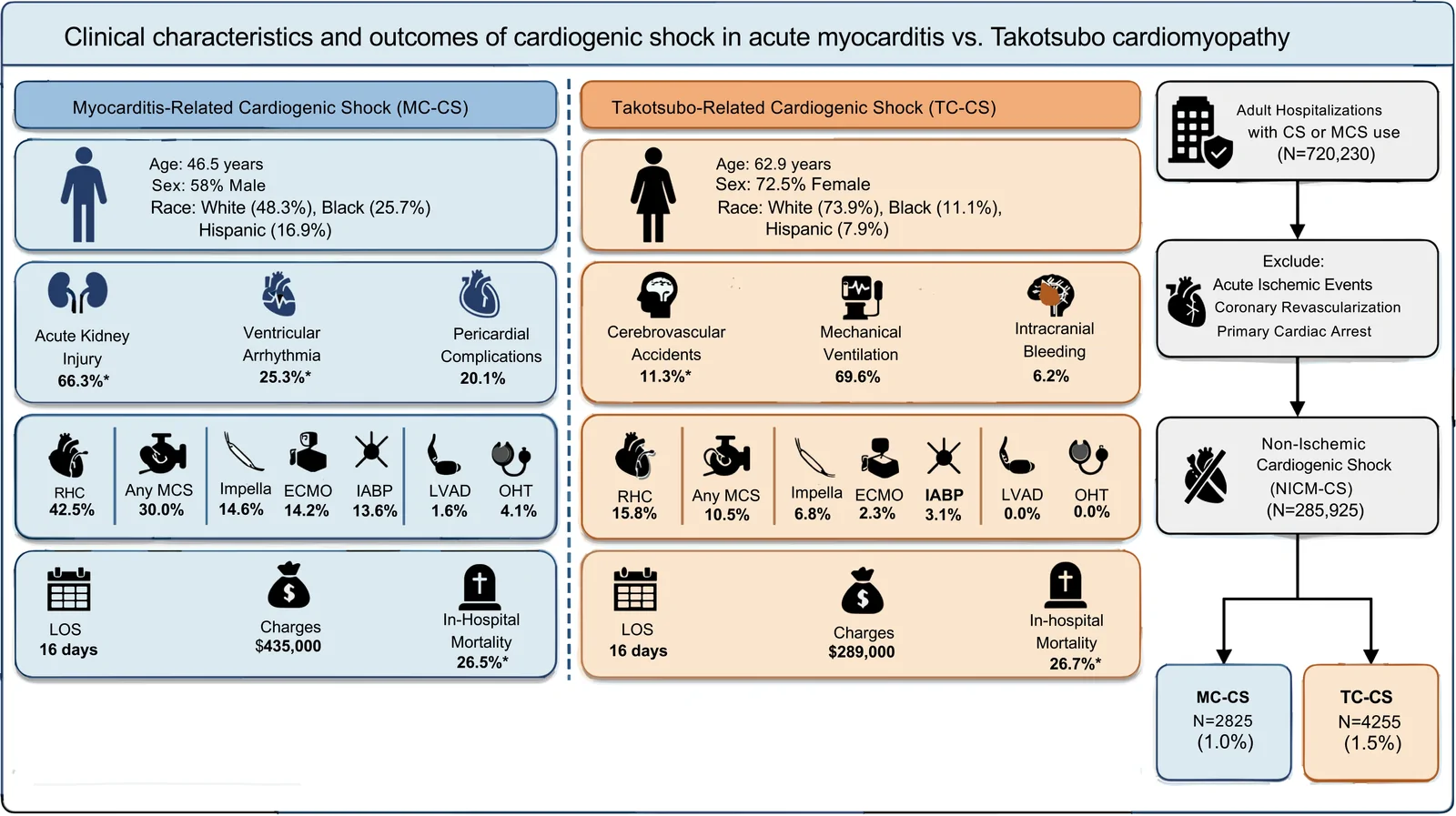
**Clinical characteristics and outcomes of cardiogenic shock in acute myocarditis vstakotsubo cardiomyopathy.** Baseline demographic features (top panel) represent unmatched, crude patient profiles to reflect real-world presentations. Clinical outcomes, in-hospital complications, and resource utilization (lower panels) reflect adjusted data following a 1:1 propensity score matching analysis. ∗Denotes a statistically nonsignificant difference between the matched cohorts (*P* > .05). Abbreviations: CS, cardiogenic shock; IABP, intra-aortic balloon pump; LOS, length of stay; LVAD, left ventricular assist device; MC-CS, myocarditis-related cardiogenic shock; MCS, mechanical circulatory support; OHT, orthotopic heart transplant; RHC, right heart catheterization; TC-CS, takotsubo cardiomyopathy-related cardiogenic shock; VA-ECMO, veno-arterial extracorporeal membrane oxygenation.

Within this nonischemic pool, MC accounted for 1.0% (n = 2825) of cases, and TC accounted for 1.5% (n = 4255). This yielded a final study population of 7080 patients, stratified into MC-CS and TC-CS cohorts. Additionally, when evaluating the broader baseline population of all hospitalizations for these 2 conditions, the overall incidence of CS was significantly higher in patients with MC compared with those with TC (8.7% vs 6.4%; *P* < .001).

### Baseline demographics and comorbidities

Before matching, patients with MC-CS were younger than those with TC-CS (46.5 ± 0.8 vs 62.9 ± 0.6 years; *P* < .001) (Table 1). Females comprised the majority of the TC-CS cohort (72.5%), whereas the MC-CS cohort was predominantly male (58.0%; *P* < .001). Racial distribution also differed; the TC-CS group had a higher proportion of White patients (73.9% vs 48.3%), whereas the MC-CS group had a higher representation of Black (25.7% vs 11.1%) and Hispanic (16.9% vs 7.9%) patients (*P* < .001). Patients with TC-CS had a higher baseline comorbidity burden, indicated by a higher mean Charlson Comorbidity Index (2.86 vs 2.38; *P* < .001). Specifically, the TC-CS group had a higher prevalence of CAD (26.9% vs 13.6%; *P* < .001), atrial fibrillation/flutter (29.4% vs 24.6%; *P* = .047), cancer (6.8% vs 4.3%), and dementia (5.5% vs 0.5%; *P* < .001). In contrast, the MC-CS cohort had a higher prevalence of obesity (21.2% vs 12.8%; *P* < .001), chronic heart failure (57.7% vs 44.3%; *P* < .001), and end-stage renal disease (5.0% vs 2.9%; *P* = .04).

**Table 1:** Baseline demographics, hospital characteristics, and pre-existing comorbidities of patients with myocarditis- vs takotsubo-related cardiogenic shock.

Variable	Before PSM			After PSM		
	Unmatched MC-CS (n = 2825)	Unmatched TC-CS (n = 4255)	Unmatched *P* value	Matched MC-CS (n = 2435)	Matched TC-CS (n = 2435)	Matched SMD
Demographics						
Age, y	46.5 ± 0.8	62.9 ± 0.6	<.001	48.1 ± 1.0	47.4 ± 1.9	0.040
Female sex	1185 (42.0%)	3085 (72.5%)	<.001	1110 (45.6%)	1080 (44.4%)	0.025
Race			<.001			
White	1363 (48.3%)	3144 (73.9%)		1250 (51.3%)	1225 (50.3%)	0.021
Black	726 (25.7%)	472 (11.1%)		555 (22.8%)	555 (22.8%)	0.000
Hispanic	477 (16.9%)	336 (7.9%)		405 (16.6%)	375 (15.4%)	0.035
Asian/Pacific Islander	161 (5.7%)	145 (3.4%)		130 (5.3%)	160 (6.6%)	0.054
Native American	11 (0.4%)	26 (0.6%)		10 (0.4%)	0 (0.0%)	0.091
Other	88 (3.1%)	132 (3.1%)		85 (3.5%)	120 (4.9%)	0.077
Primary payer			<.001			
Medicare	648 (23.0%)	2408 (56.6%)		570 (23.4%)	645 (26.5%)	0.068
Medicaid	743 (26.3%)	787 (18.5%)		625 (25.7%)	695 (28.5%)	0.066
Private insurance	1265 (44.8%)	953 (22.4%)		1000 (41.1%)	830 (34.1%)	0.146
Self-pay	169 (6.0%)	108 (2.6%)		135 (5.5%)	130 (5.3%)	0.010
Median income			.76			
Q1 (lowest)	761 (26.9%)	1087 (25.5%)		660 (27.1%)	640 (26.3%)	0.019
Q2	761 (26.9%)	1102 (25.9%)		670 (27.5%)	640 (26.3%)	0.028
Q3	715 (25.3%)	1087 (25.5%)		595 (24.4%)	685 (28.1%)	0.085
Q4 (highest)	588 (20.8%)	980 (23.0%)		510 (20.9%)	470 (19.3%)	0.040
Hospital characteristics						
Hospital region			.06			
Northeast	590 (20.9%)	910 (21.4%)		505 (20.7%)	465 (19.1%)	0.041
Midwest	689 (24.4%)	1136 (26.7%)		610 (25.1%)	560 (23.0%)	0.048
South	1000 (35.4%)	1221 (28.7%)		840 (34.5%)	910 (37.4%)	0.060
West	545 (19.3%)	991 (23.3%)		480 (19.7%)	500 (20.5%)	0.020
Teaching status			<.001			
Urban teaching	2585 (91.5%)	3566 (83.8%)		2205 (90.6%)	2255 (92.6%)	0.071
Urban nonteaching	195 (6.9%)	540 (12.7%)		185 (7.6%)	120 (4.9%)	0.107
Rural	45 (1.6%)	149 (3.5%)		45 (1.8%)	60 (2.5%)	0.039
Hospital bed size			<.001			
Small	215 (7.6%)	495 (11.6%)		180 (7.4%)	140 (5.7%)	0.065
Medium	475 (16.8%)	962 (22.6%)		465 (19.1%)	495 (20.3%)	0.030
Large	2135 (75.6%)	2800 (65.8%)		1790 (73.5%)	1800 (73.9%)	0.009
Comorbidities						
Charlson comorbidity index	2.38 ± 0.08	2.86 ± 0.08	<.001	2.41 ± 0.10	2.51 ± 0.20	0.043
CHF	1630 (57.7%)	1885 (44.3%)	<.001	1365 (56.1%)	1350 (55.4%)	0.012
CAD	384 (13.6%)	1145 (26.9%)	<.001	360 (14.8%)	355 (14.6%)	0.005
Prior PCI	51 (1.8%)	123 (2.9%)	.17	50 (2.1%)	30 (1.2%)	0.064
Prior CABG	20 (0.7%)	60 (1.4%)	.23	15 (0.6%)	15 (0.6%)	0.000
Prior MI	71 (2.5%)	226 (5.3%)	.009	70 (2.9%)	65 (2.7%)	0.011
Prior ICD	116 (4.1%)	51 (1.2%)	<.001	65 (2.7%)	20 (0.8%)	0.133
Atrial fibrillation/flutter	695 (24.6%)	1251 (29.4%)	.047	590 (24.2%)	590 (24.2%)	0.000
Diabetes mellitus	345 (12.2%)	540 (12.7%)	.80	325 (13.3%)	425 (17.5%)	0.120
CKD	511 (18.1%)	723 (17.0%)	.62	400 (16.4%)	365 (15.0%)	0.040
ESRD	141 (5.0%)	123 (2.9%)	.04	125 (5.1%)	180 (7.4%)	0.094
Cancer	121 (4.3%)	289 (6.8%)	.05	115 (4.7%)	155 (6.4%)	0.066
Prior stroke	130 (4.6%)	272 (6.4%)	.16	95 (3.9%)	105 (4.3%)	0.019
Dementia	14 (0.5%)	234 (5.5%)	<.001	15 (0.6%)	25 (1.0%)	0.036
Obesity	599 (21.2%)	545 (12.8%)	<.001	475 (19.5%)	510 (20.9%)	0.038
PVD	232 (8.2%)	284 (6.7%)	.46	200 (8.2%)	190 (7.8%)	0.016

Data shown before and after propensity score matching. Values are presented as weighted absolute numbers (n) and percentages (%) or mean ± standard error (SE). Standardized mean difference (SMD) represents the degree of balance between covariates in the matched cohort, with a value <0.10 indicating adequate balance.

CABG, coronary artery bypass grafting; CAD, coronary artery disease; CCI, Charlson Comorbidity Index; CHF, congestive heart failure; CKD, chronic kidney disease; ESRD, end-stage renal disease; ICD, implantable cardioverter-defibrillator; MC-CS, myocarditis-related cardiogenic shock; MI, myocardial infarction; PCI, percutaneous coronary intervention; PSM, propensity score matching; PVD, peripheral vascular disease; SE, standard error; SMD, standardized mean difference; TC-CS, takotsubo cardiomyopathy-related cardiogenic shock.

Propensity score matching yielded 2435 balanced pairs (all SMD <0.10). In the matched cohort, the mean Charlson Comorbidity Index (2.41 ± 0.10 vs 2.51 ± 0.20; SMD 0.043) and individual comorbidities were comparable between groups (Table 1).

### MCS utilization and in-hospital outcomes

In the matched cohort, patients with MC-CS required significantly higher rates of advanced mechanical support. Utilization of any temporary MCS was approximately 3-fold higher in the MC-CS group compared with the TC-CS group (30.0% vs 10.5%; *P* < .001), including IABP (13.6% vs 3.1%; *P* = .003), Impella (14.6% vs 6.8%; *P* = .04), and VA-ECMO (14.2% vs 2.3%; *P* = .003). Advanced therapies, including left vetricular assist device (1.6% vs 0.0%; *P* = .02) and orthotropic heart transplantation (4.1% vs 0.0%; *P* < .001), were also more frequent in the MC-CS cohort. Furthermore, patients with MC-CS were more likely to undergo invasive hemodynamic monitoring via RHC (42.5% vs 15.8%; *P* < .001) (Table 2).

**Table 2:** Clinical profiles, resource utilization, and in-hospital complications of patients with myocarditis- vs takotsubo-related cardiogenic shock

Variable	Before PSM			After PSM		
	Unmatched MC-CS (n = 2825)	Unmatched TC-CS (n = 4255)	Unmatched *P* value	Matched MC-CS (n = 2435)	Matched TC-CS (n = 2435)	Matched *P* value
In-hospital mortality	720 (25.5%)	1421 (33.4%)	.003	645 (26.5%)	650 (26.7%)	.97
Length of stay, d	16.4 ± 0.8	12.7 ± 0.4	<.001	16.2 ± 1.0	16.0 ± 2.6	.96
Total charges, USD	466,146 ± 32,651	241,304 ± 10,488	<.001	435,121 ± 37,716	288,522 ± 44,323	.008
Discharged to SNF/rehab	545 (19.3%)	1120 (26.3%)	<.001	465 (19.1%)	565 (23.2%)	.36
MCS utilization						
Any temporary mechanical support	905 (32.0%)	417 (9.8%)	<.001	730 (30.0%)	255 (10.5%)	<.001
Impella	449 (15.9%)	191 (4.5%)	<.001	355 (14.6%)	165 (6.8%)	.04
IABP	370 (13.1%)	200 (4.7%)	<.001	330 (13.6%)	75 (3.1%)	.003
VA-ECMO	449 (15.9%)	81 (1.9%)	<.001	345 (14.2%)	55 (2.3%)	.003
LVAD	71 (2.5%)	9 (0.2%)	<.001	40 (1.6%)	0 (0.0%)	.02
Heart transplant	121 (4.3%)	0 (0.0%)	<.001	100 (4.1%)	0 (0.0%)	<.001
In-hospital complications						
Mechanical ventilation	1164 (41.2%)	2225 (52.3%)	<.001	990 (40.7%)	1695 (69.6%)	<.001
RHC	1195 (42.3%)	740 (17.4%)	<.001	1035 (42.5%)	385 (15.8%)	<.001
Ventricular arrhythmia	754 (26.7%)	774 (18.2%)	<.001	615 (25.3%)	530 (21.8%)	.51
ICD placement	124 (4.4%)	81 (1.9%)	.005	105 (4.3%)	45 (1.9%)	.28
Acute liver injury	904 (32.0%)	851 (20.0%)	<.001	740 (30.4%)	710 (29.2%)	.82
Acute kidney injury	1978 (70.0%)	2340 (55.0%)	<.001	1615 (66.3%)	1395 (57.3%)	.11
Cerebrovascular accidents	169 (6.0%)	400 (9.4%)	.02	145 (6.0%)	275 (11.3%)	.06
Intracranial bleeding	40 (1.4%)	170 (4.0%)	.005	25 (1.0%)	150 (6.2%)	.008
Procedural bleeding	40 (1.4%)	17 (0.4%)	.03	35 (1.4%)	20 (0.8%)	.60
Pericardial complications	557 (19.7%)	238 (5.6%)	<.001	490 (20.1%)	205 (8.4%)	.04

Data shown before and after propensity score matching. Categorical variables are presented as frequencies and percentages n (%); continuous variables are presented as mean ± standard error (SE).

IABP, intra-aortic balloon pump; ICD, implantable cardioverter-defibrillator; LVAD, left ventricular assist device; MC-CS, myocarditis-related cardiogenic shock; MCS, mechanical circulatory support; RHC, right heart catheterization; SNF, skilled nursing facility; TC-CS, takotsubo cardiomyopathy-related cardiogenic shock; USD, United States dollars; VA-ECMO, veno-arterial extracorporeal membrane oxygenation.

Although the incidence of pericardial complications was higher in the MC-CS cohort (20.1% vs 8.4%; *P* = .04), the TC-CS cohort had significantly higher rates of invasive mechanical ventilation (69.6% vs 40.7%; *P* < .001) and intracranial bleeding (6.2% vs 1.0%; *P* = .008). Other complications were comparable between groups, including acute kidney injury (66.3% vs 57.3%; *P* = .11), acute liver injury (30.4% vs 29.2%; *P* = .82), ventricular arrhythmias (25.3% vs 21.8%; *P* = .51), new implantable cardioverter defib implantation (4.3% vs 1.9%; *P* = .28), and cerebrovascular accidents (6.0% vs 11.3%; *P* = .06) (Table 2).

### Mortality, resource utilization, and disposition

Prior to propensity score matching (PSM), unadjusted in-hospital mortality was significantly higher in the TC-CS cohort compared with MC-CS (33.4% vs 25.5%; OR, 1.46; 95% CI, 1.14-1.88; *P* = .003). However, in the matched cohort, in-hospital mortality was comparable between the MC-CS and TC-CS groups (26.5% vs 26.7%; *P* = .97). Although the mean length of stay was similar (16.2 ± 1.0 vs 16.0 ± 2.6 days; *P* = .96), patients with MC-CS incurred significantly higher total hospital charges ($435,121 ± 37,716 vs $288,522 ± 44,323; *P* = .008). Discharge to skilled nursing or rehabilitation facilities was also comparable between the 2 groups (19.1% vs 23.2%; *P* = .36).

## Discussion

To our knowledge, this is the first study to directly compare clinical characteristics, MCS utilization, and in-hospital outcomes of MC-CS vs TC-CS. Several observations emerged from this analysis. First, the cohorts exhibited divergent demographic profiles; MC-CS predominantly involved younger males, whereas TC-CS primarily affected older females. Second, the utilization of temporary MCS and RHC was approximately 3-fold higher in MC-CS compared with TC-CS. Third, MC-CS was associated with a more resource-intensive hospital course, whereas length of stay was comparable between groups; patients with MC-CS incurred significantly higher total hospital charges. Fourth, although crude in-hospital mortality was higher in the TC-CS cohort, mortality rates and discharge disposition were comparable after PSM balanced baseline demographics and comorbidities.

Consistent with prior literature, the MC-CS cohort predominantly comprised younger males and exhibited a more diverse racial distribution (48% White, 26% Black).^13^ In contrast, TC-CS affected older, postmenopausal women and a predominantly White demographic (74% White, 11% Black).^6^^,^^11^ Although female sex has been reported to be associated with a higher relative risk of cardiogenic shock among patients with myocarditis, men remain more frequently represented in MC-CS cohorts, likely reflecting the higher baseline prevalence of myocarditis in males.^11^^,^^13^, ^14^, ^15^, ^16^ In the present study, sex was not independently associated with in-hospital mortality in either cohort.

Cardiogenic shock has been reported to complicate approximately 10% of patients with MC and TC. Although the administrative constraints of the NIS preclude direct risk stratification by Society for Cardiovascular Angiography and Interventions shock stage, the overall propensity-matched in-hospital mortality of approximately 26% observed in our cohorts aligns with contemporary nonischemic clinical registries.^8^, ^9^, ^10^, ^11^ In the present study, despite comparable baseline demographics and comorbidity burden following PSM (Table 1), patients with MC-CS experienced a more resource-intensive clinical course. This pattern is consistent with fulminant myocarditis, a phenotype characterized by diffuse myocardial inflammation, myocyte injury, and interstitial edema leading to acute hemodynamic compromise.^5^^,^^16^, ^17^, ^18^ The severity of myocardial dysfunction in MC-CS likely drives the higher rates of pericardial complications and the significantly greater requirement for invasive hemodynamic monitoring, MCS, and heart transplant observed in this cohort.

In contrast, the TC-CS cohort demonstrated a distinct complication profile, marked by a greater need for invasive mechanical ventilation and higher rates of neurologic events, including intracranial hemorrhage and a trend toward higher cerebrovascular accidents (11.3% vs 6.0%, *P* = .06). These findings align with prior observations linking TC to acute neurologic triggers and thromboembolic complications arising from severe, transient left ventricular apical ballooning and blood stasis.^19^^,^^20^

The utilization of temporary MCS has increased in recent years for the management of CS, as it effectively decreases myocardial workload while improving systemic perfusion, coronary perfusion, and ventricular offloading.^21^^,^^22^ These devices serve as a bridge to recovery, decision, further support, or transplantation. In our PSM cohort, temporary MCS was used in 30% and 10.5% of patients with MC-CS and TC-CS, respectively.

Early MCS in MC-CS provides vital hemodynamic stability and is associated with acceptable intermediate-term survival. Diffuse inflammatory myocardial involvement frequently causes biventricular failure, making isolated univentricular support insufficient. Biventricular support, such as VA-ECMO combined with Impella or biventricular assist devices, is frequently required to decompress the left vectricle and promote myocardial recovery.^2^^,^^12^^,^^16^^,^^22^ In the MC-CS cohort, 42% of patients treated with VA-ECMO also received concomitant Impella support.

In TC-CS, exogenous vasopressors and inotropes are relatively contraindicated. They share a causative association with the catecholamine-driven pathogenesis of the syndrome and increase basal hypercontractility, precipitating or exacerbating dynamic left ventricular outflow tract obstruction.^22^, ^23^, ^24^ Early MCS offers a safer hemodynamic bridge for spontaneous left ventricle recovery, avoiding further catecholamine toxicity. Additionally, afterload reduction with IABP may further worsen left ventricular outflow tract obstruction in susceptible patients, underscoring the importance of careful patient selection and close echocardiographic monitoring.^2^^,^^22^^,^^25^

In conclusion, CS secondary to MC and TC represents 2 distinct clinical phenotypes with divergent management requirements. MC-CS is characterized by a high intensity of resource utilization, including a nearly 3-fold higher requirement for temporary MCS and RHC, resulting in greater total hospital charges. Conversely, TC-CS demonstrates a higher burden of neurologic complications and a greater requirement for invasive mechanical ventilation. Following adjustment for baseline differences, in-hospital mortality and discharge disposition were comparable between the 2 etiologies. These findings may suggest that the observed crude mortality gap is primarily influenced by patient age and baseline comorbidities rather than the shock etiology itself.

### Limitations

Several limitations inherent to the retrospective, observational design of this administrative database study warrant consideration. First, reliance on ICD-10-CM coding for cohort identification and procedural mapping introduces inherent risks of misclassification, underreporting, and administrative billing inaccuracies. This is particularly relevant for TC, given its potential phenotypic overlap with acute coronary syndrome, and for acute myocarditis, which lacks absolute diagnostic specificity without endomyocardial biopsy or cardiac magnetic resonance imaging. Despite implementing stringent exclusion criteria for acute coronary syndrome and coronary revascularization, residual misclassification bias cannot be entirely excluded (Supplemental Table S1 details all used ICD-10-CM codes). Second, the NIS fundamentally lacks granular patient-level clinical, echocardiographic, and physiological data, including left ventricular ejection fraction, left ventricular outflow tract gradients, laboratory markers of tissue hypoperfusion (e.g., serum lactate), and specific invasive hemodynamic indices. The dataset precludes stratification by Society for Cardiovascular Angiography and Interventions shock stages, which is a critical determinant of cardiogenic shock risk stratification and mortality prediction. Third, the absence of pharmacological data prevents the assessment of specific vasoactive or inotropic requirements, which carry divergent therapeutic implications in myocarditis compared with takotsubo syndrome. Fourth, although PSM was employed to adjust for baseline imbalances, the potential for unmeasured confounding persists. Finally, the NIS is restricted to index hospitalizations, precluding longitudinal evaluation postdischarge.

## CRediT authorship contribution statement

**Hossam Ibrahim:** Writing – review & editing, Writing – original draft, Visualization, Methodology, Formal analysis, Data curation, Conceptualization. **Oluchi Ndulue:** Writing – original draft. **Fatmaelzahraa Badr:** Writing – original draft, Visualization, Data curation. **Valentine C. Nriagu:** Writing – original draft. **Hasan Alarouri:** Writing – review & editing. **Armando Seitllari:** Writing – review & editing. **Binit Aryal:** Writing – review & editing. **Supraja Achuthanandan:** Writing – review & editing. **Steven Sorci:** Writing – review & editing, Conceptualization. **Bilal Malik:** Writing – review & editing. **Gerald Hollander:** Writing – review & editing. **Robert Frankel:** Writing – review & editing. **Jacob Shani:** Writing – review & editing. **Vijay Shetty:** Writing – review & editing, Supervision, Methodology, Conceptualization.

## Footnotes

To access the supplemental material accompanying this article, visit the online version of the *Journal of the Society for Cardiovascular Angiography & Interventions* at 10.1016/j.jscai.2026.105460.
