# Supplementary material for: Clinical Characteristics and Outcomes of Cardiogenic Shock in Acute Myocarditis Versus Takotsubo Cardiomyopathy: A Propensity Matched Nationwide Analysis
Source: J Soc Cardiovasc Angiogr Interv. 2026 Jun 18;5(9):105460. doi: 10.1016/j.jscai.2026.105460 (PMC13591534; doi:10.1016/j.jscai.2026.105460)
Supplement: Supplementary file 1 [file mmc1.docx]

### **Supplementary Table S1: International classification of diseases, tenth revision, clinical modification (ICD-10-CM) and procedure coding system (ICD-10-PCS) codes used to define clinical conditions, baseline comorbidities, and in-hospital interventions.**

| **Variable / Clinical Condition** | **ICD-10 Codes** |
| --- | --- |
| Cardiogenic shock | R57.0 |
| Myocarditis | I40.x, I41, B33.22, B33.24, B26.82, J10.82, J11.82, I51.4 |
| Takotsubo cardiomyopathy | I51.81 |
|  |  |
| Comorbidities | |
| Chronic heart failure | I09.81, I11.0, I13.0, I13.2, I50.1, I50.20, I50.22, I50.30, I50.32, I50.40, I50.42, I50.810, I50.812, I50.814, I50.82, I50.83, I50.84, I50.89, I50.9 |
| End-stage renal disease | N18.6, Z99.2 |
| History of PCI | Z95.5, Z98.61 |
| History of CABG | Z95.1 |
| History of MI | I25.2 |
| History of CAD | I25.x |
| History of Atrial fibrillation/flutter | I48.x |
| History of ICD | Z95.810 |
| Dementia | F00.x–F03.x, F05.1, G30.x, G31.1 |
| Diabetes mellitus | E10.0, E10.1, E10.9, E11.0, E11.1, E11.9,  E12.0, E12.1, E12.9, E13.0, E13.1, E13.9,  E14.0, E14.1, E14.9, E10.2–E10.8, E11.2–E11.8, E12.2–E12.8, E13.2–E13.8, E14.2–E14.8 |
| Cancer | C00.x–C26.x, C30.x–C34.x, C37.x–C41.x,  C43.x, C45.x–C58.x, C60.x–C76.x, C81.x–  C85.x, C88.x, C90.x–C97.x, C77.x–C80.x |
| In-hospital complications & interventions | |
| Mechanical ventilation (>24 hours) | 5A1945Z, 5A1955Z |
| Ventricular arrhythmia | I47.2, I49.01, I49.02 |
| Cerebrovascular accident | I63.x, I64.x, G45.x, I60.x , I61.x , I62.x |
| Cardiac arrest | I46.x |
| Acute myocardial infarction | I21.x, I22.x |
| Acute kidney injury | N17.0, N17.1, N17.2, N17.8, N17.9 |
| Acute liver injury | R7401, R748, K720x |
| Intra-aortic balloon pump | 5A02110, 5A02210 |
| Impella (Micro-axial flow pump) | 5A0221D, 02HA3RZ, 02HA0RZ, 02HA3RJ, 02HA0RJ, 02HA3RS, 02HA0RS, 02PA3RZ, 02PA0RZ, 02PA3RS, 02PA0RS |
| VA-ECMO | 5A1522G, 5A1522F |
| LVAD | 02HA0QZ |
| Orthotopic heart transplant | 02YA0Z0 |
| Right heart catheterization | 02HP30Z, 02HP32Z, 02HQ30Z, 02HQ32Z, 02HR30Z, 4A0239Z, 4A023N6, 4A023N8, 4A1239, 4A133B3 |
| Percutaneous Coronary Intervention (PCI) | 02103, 02113, 02123, 02133, 02703, 02704, 02713, 02714, 02723, 02724, 02733, 02734, 02C03, 02C04, 02C13, 02C14, 02C23, 02C24, 02C33, 02C34, 02F03, 02F04, 02F13, 02F14, 02F23, 02F24, 02F33, 02F34, 02N03, 02N04, 02N13, 02N14, 02N23, 02N24, 02N33, 02N34, 02Q03, 02Q04, 02Q13, 02Q14, 02Q23, 02Q24, 02Q33, 02Q34, 02U03, 02U04, 02U13, 02U14, 02U23, 02U24, 02U33, 02U34, X2C03, X2C04, X2C13, X2C14, X2C23, X2C24, X2C33, X2C34 |
| CABG | 0210x, 0211x, 0212x, 0213x |
| ICD placement | 0JH608, 0JH609, 0JH638, 0JH639 |
| Intracranial bleeding | I60.x, I61.x, I62.x |
| Procedural bleeding | I97.4, I97.6, T82.83 |
| Pericardial complications | I31.2, I31.39, I31.4 |

### The "x" placeholder indicates that all sub-classifications under that specific parent billing code were included in the definition. Abbreviations**:** CABG, coronary artery bypass grafting; CAD, coronary artery disease; ICD, implantable cardioverter-defibrillator; LVAD, left ventricular assist device; MI, myocardial infarction; PCI, percutaneous coronary intervention; RHC, right heart catheterization; VA-ECMO, veno-arterial extracorporeal membrane oxygenation.
